# Perspectives on Open Science and The Future of Scholarly Communication: Internet Trackers and Algorithmic Persuasion

**DOI:** 10.3389/frma.2021.748095

**Published:** 2021-12-23

**Authors:** Tiberius Ignat, Paul Ayris, Beatrice Gini, Olga Stepankova, Deniz Özdemir, Damla Bal, Yordanka Deyanova

**Affiliations:** ^1^ Scientific Knowledge Services, Munich, Germany; ^2^ LCCOS - Library, Culture, Collections, Open Science, University College London, London, United Kingdom; ^3^ Cambridge University Library (CUL), University of Cambridge, Cambridge, United Kingdom; ^4^ CIIRC (Czech Institute of Informatics and Robotics and Cybernetics), BEAT (Biomedical Engineering and Assited Technologies) Department, Czech Technical University in Prague, Prague, Czechia

**Keywords:** scholarly communication, track, persuade, readers, authors, open science, trust, infrastructure

## Abstract

The current digital content industry is heavily oriented towards building platforms that track users’ behaviour and seek to convince them to stay longer and come back sooner onto the platform. Similarly, authors are incentivised to publish more and to become champions of dissemination. Arguably, these incentive systems are built around public reputation supported by a system of metrics, hard to be assessed. Generally, the digital content industry is permeable to non-human contributors (algorithms that are able to generate content and reactions), anonymity and identity fraud. It is pertinent to present a perspective paper about early signs of track and persuasion in scholarly communication. Building our views, we have run a pilot study to determine the opportunity for conducting research about the use of “track and persuade” technologies in scholarly communication. We collected observations on a sample of 148 relevant websites and we interviewed 15 that are experts related to the field. Through this work, we tried to identify 1) the essential questions that could inspire proper research, 2) good practices to be recommended for future research, and 3) whether citizen science is a suitable approach to further research in this field. The findings could contribute to determining a broader solution for building trust and infrastructure in scholarly communication. The principles of Open Science will be used as a framework to see if they offer insights into this work going forward.

## Introduction

Open Science is part of the “new normal” as the world emerges from the covid-19 pandemic. Open Access to publications is now a well-developed phenomenon for research outputs.

In Europe, there are eight themes which are commonly seen to be part of Open Science principle and practice, including *Research Integrity* and *The Future of Scholarly Communication*, both being the subject of our perspective paper.

These are: 1) Rewards and Incentives, 2) Indicators and Next-Generation Metrics, 3) Future of Scholarly Communication, 4) European Open Science Cloud (EOSC), 5) FAIR data, 6) Research Integrity, 7) Skills and Education, 8) Citizen Science (Open Science EU, 2020).

Research Integrity comprises a set of principles which should underpin research practice. As the 23 research-intensive universities of LERU concluded in their report Open Science and its role in universities: a roadmap for cultural change (Ayris et al., 2018a), a move to Open Science represents a fundamental cultural shift for researchers. The ALLEA code on Research Integrity states that good research practices are based on fundamental principles of research integrity, these being: Reliability, Honesty, Respect, Accountability (ALLEA, 2017a).

ALLEA (ALLEA, 2017b) has produced the European Code of Conduct for Research Integrity that addresses challenges emanating from technological developments and social media, among other areas. For example, it says that “Researchers, research institutions and organisations [should] provide transparency about how to access or make use of their data and research materials.” As such, it is recognised by the European Commission as the reference document for research integrity for all EU-funded research projects and as a model for organisations and researchers across Europe.

Web trackers enable profitable business models for organisations that develop web-based applications, especially for those that interfere with people’s behaviour. In some cases, governmental agencies use such models, too. Some tech companies consider these trackers fundamental for “the free and open” Internet as we know it (BBC News, 2021). We disagree with this model for developing the Internet and its role in society. Furthermore, we consider this an inappropriate model for the field of scholarly communication.

While allowing ourselves to be surveilled by unknown organisations in exchange for free or underpriced services (Barbu, 2014), we develop a new culture in which our society is trading hard-won freedom for questionable prosperity. That culture will be inherited by future generations, who will be challenged to change it when this trade-off will no longer be bearable.

This paper presents a set of recommendations and the authors’ perspective on using modern technologies in scholarly communication processes. To build our views, we studied 148 web pages related to the field and we collected 15 expert opinions.

## Observations and Discussion

Modern technologies based on tracking (in Internet and mobile applications), including Artificial Intelligence (AI), digital persuasive technologies and—to an extent—Robotic Process Automation (RPA), are common elements in the new landscape of content creation, content management and information. Scientific knowledge and scholarly communication could become the new territory to be infested by these tracking-related technologies.

While some trackers are less invasive and are placed to support basic functionalities for websites and apps, most trackers are used to expose our behaviour and personal data, for the benefit of a small group of organisations. They are used in prediction models that fuel the business of recommendation engines (Beckers, 2021). They contribute to a surveillance economy and are used to create individual psychographic profiles (Gibney, 2018).

Both desktop and mobile versions of web tracking are implemented by utilizing a plethora of tracking technologies, including cookies, JavaScript components, local shared objects, iframes, and relying on the technology of third-party trackers (Mittal, 2010). The most common way to prevent cookie tracing is to configure the internet browser configuration in order to block third-party cookies. Browser extensions on the other hand could be of assistance in this case, and Incognito mode (which is also referred to as private browsing) can additionally offer protection as well, though not disabling third-party cookies completely (Bielova, 2017). Consequently, a privacy scoring model for each website to evaluate the privacy risks could give detailed insights for detection (Hamed and Ayed, 2015).

The future of tracking shall be evaluated in accordance with the new Internet protocols, passive network traffic monitoring and Developers’ technical blogs since a variety of information can be gathered by the analysis of new protocols and extensions covering different web standards and their functionalities respectively (Bujlow et al., 2017). Forrester’s data security and privacy playbook provides the tools, information and analysis to aid with the protection of data privacy abuse with a framework that has a three-step process (Balaouras, 2019): ensuring the necessities for better data security and privacy, implementing a road map to brace the business and enhance data security and privacy and carrying out security and privacy solutions, thus affirming the execution of the privacy of data (Abdullah, 2020).

**FIGURE 1:**
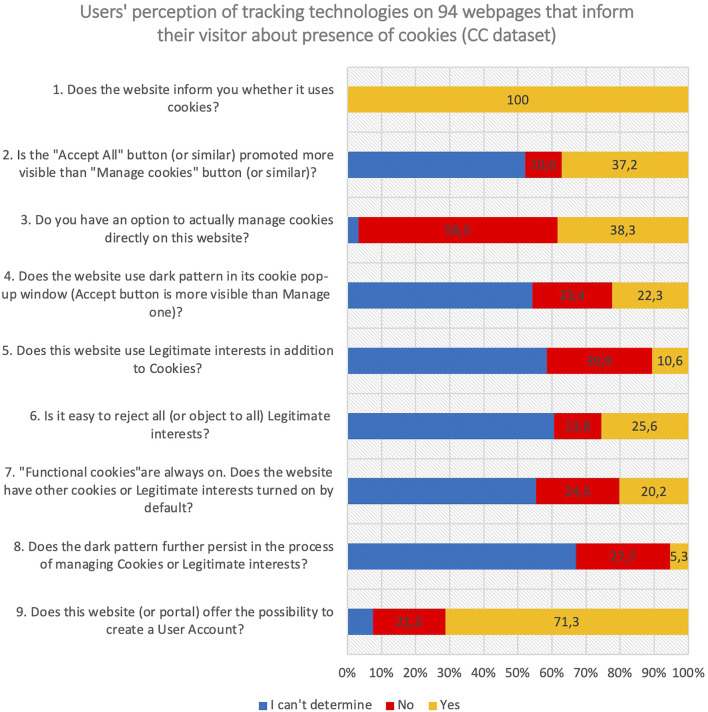
User´s perception of tracking technologies on the subset **CC** described in the text.

To investigate the frequency of tracking in scholarly communication, we analysed 148 web pages related to scholarly communication. They represent a mix of publishers (55), technology companies (35), preprint servers (27), content aggregators (24), libraries and others (7). They answered 9 questions (Figure 1).

### Quantitative Observations

The answer to the first question “Does the website inform you whether it uses cookies?” divides the original dataset into 2 disjunctive subsets—the **CC** dataset with web pages openly confirming use of cookies (*n* = 94) and the **NC** dataset with web pages that don’t mention cookies (*n* = 54). To verify the use of cookies in the **NC** dataset, we checked it with cookie management applications, revealing cookies’ presence in most of them. This suggests needed improvement.

Questions 2–8 are not relevant for websites in the **NC** dataset, hence the detailed analysis is focussed to the **CC** dataset containing 94 web pages.

The results highlight surprising observations:

• 60% of webpages in **CC** subset offer no option to manage cookies (Q3). Even if this option is presented, the “Accept All” button is promoted more visibly often (37,2%, see Q2).
• Questions 2 and 4–8 are answered “I can’t determine” in more than 50% of cases – suggesting that managing cookies is far from intuitive.

While of the original set of 148 webpages, 68,9% offer the possibility to create user accounts, this percentage is 71,3% for the **CC** dataset. In psychographic profiling, data collected through user accounts is usually complementary to the data collected through trackers (Aries Systems, 2020; art. 2 and art. 7), with potential for the de-anonymization of the datasets.

The dataset could be downloaded from here: https://doi.org/10.5281/zenodo.5139523.

### Expert Interviews

**FIGURE 2:**
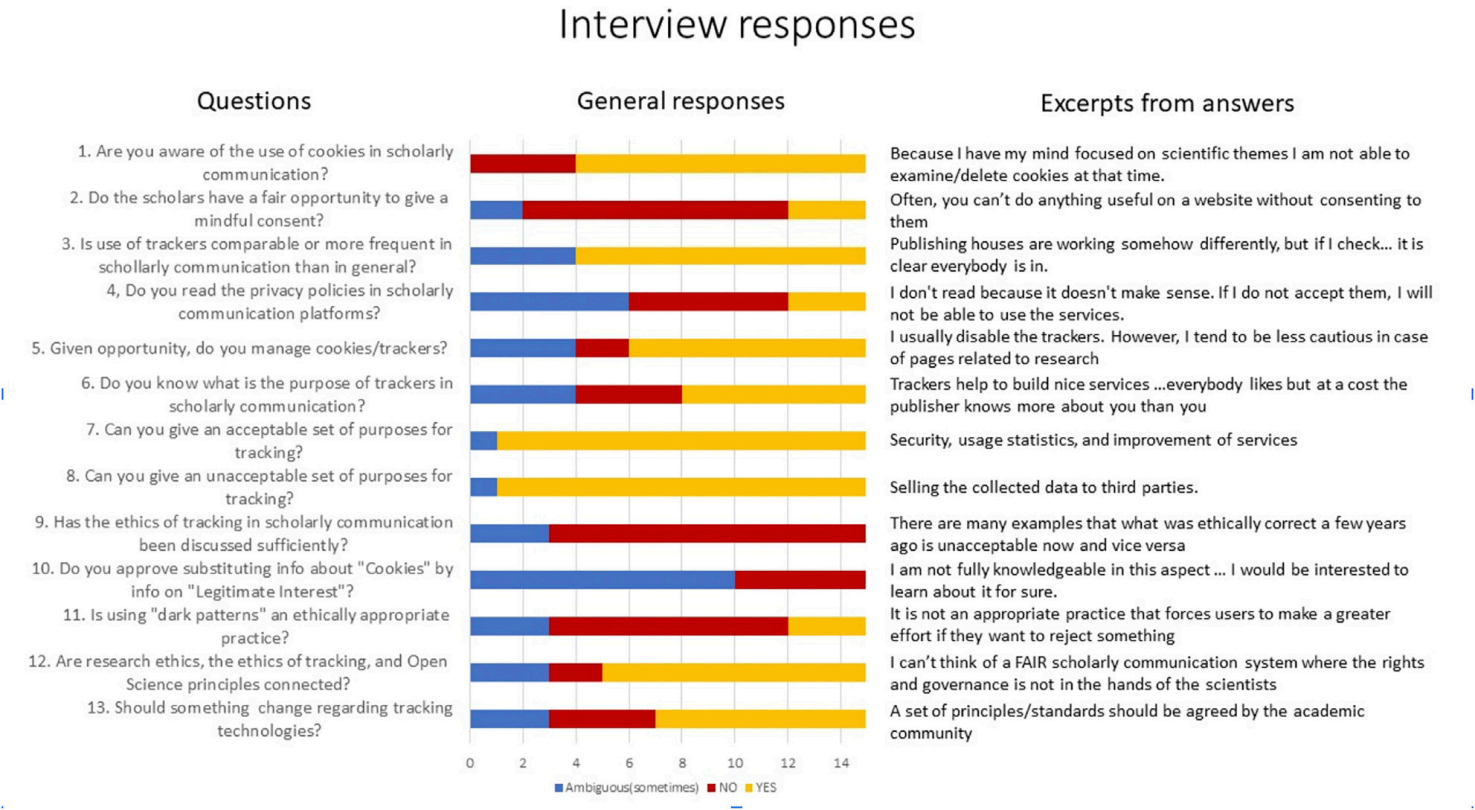
The result of interviewing 15 experts of scholarly communication.

To understand subjective experiences of trackers in scholarly communication, we conducted written interviews with fifteen experts in the fields of scholarly communication. They were selected based on the authors’ professional networks. While this cannot be considered a representative sample, it can provide an initial insight into the community’s perceptions of these issues. A summary of their answers to 13 questions is presented in Figure 2 with both an overview of responses and selected quotations.

The overview bar chart interprets the answers using 3 values: NO, YES or “Ambiguous” corresponding to “I do not know” (questions 2,3 6 and 9), “Rarely” (questions 4 and 5), no answer (questions 7 and 8) or “I am not sure—I do not know enough about the topic to give a clear answer” (questions 10–13). This rough simplification of answers is used here to highlight the extent of interest in the topic among the academic community. The graph confirms that scholars believe they do not have fair opportunities to give mindful consent for tracking (question 2) and that the ethics of tracking should be discussed more extensively in the academic community (question 9). Moreover, differing opinions about acceptable and unacceptable purposes of the technology suggest that fruitful debates could be organised if the right forums were created.

### Essential Questions for the Scholarly Communication Community

Scholarly communication community might be tempted to use trackers and persuasive technologies. Some might serve the interest of readers, authors, peer-reviewers, and research organisations.

Hence, it is important to identify what questions need answers before these technologies become the norm.

We believe these questions are essential for the members of scholarly communication community when considering to use modern technologies like web tracking, AI and RPA:

1. What are the highest ethical paths for a field of communication that needs to build trust and communicate evidence and knowledge?
2. What vulnerabilities are brought to the research community?
3. What are the real opportunities for researchers and for society?
4. What is realistic and what is utopic in these technologies? Which are the demonstrated positive effects of those technologies?
5. How can we ensure that those technologies develop human-centric?
6. Who is governing the development of those technologies?
7. What system could guard the researchers from being manipulated by such technologies (Michael, 2019)?
8. What is the impact of such technologies on educating next generations of curious minds?

## Authors’ Perspective

Our analysis showed that only 64% of websites inform users of their use of cookies, despite this being a legal requirement in the EU, where we accessed them. Even worse, cookies appear in most of 54 websites from the NC dataset consisting of those websites that don’t provide the visitor with any information about their cookies policy. The option to manage cookies was either lacking or disguised with “dark patterns” in the majority of sites, contrary to our expectations for transparency and freedom in internet use. Moreover, 69% of all studied websites offered the option to create an account, even though the benefits to users were not always evident. User accounts can store large amounts of information and could be combined with cookie data to track and manipulate behaviour. This paints a troubling picture of the state of tracking in scholarly communication: there is little transparency and significant potential for persuasive technologies to become commonplace.

The experts’ interviews corroborated this lack of transparency: most interviewees assumed that large amounts of data were being collected, but admitted to having a poor understanding of what the process and aims were. They also indicated that, although the option to manage cookies exists in principle, in reality most cookies are accepted unquestioningly due to difficulties and time required to manage them manually. Most concerning was the fact that several interviewees instinctively trust scholarly communication platforms, saying for instance: “I usually disable the trackers. However, I tend to be less cautious in the case of pages related to research as I hope there is a smaller risk of misuse of this data. Of course, I have no hard data supporting this assumption.” Thus scholarly communication platforms may be benefitting from a greater degree of trust from their users, but not setting higher standards for themselves, compared to other websites.

Interviewees identified some beneficial uses of tracking, namely personalised recommendations for reading materials, conferences and job opportunities, and the collection of anonymised data to improve website design or report usage statistics. On the other hand, the selling of personal data was overwhelmingly cited as an unacceptable use of tracking. Other unacceptable uses included the profiling of users based on protected characteristics such as ethnicity or political affiliation, advertising (although not unanimously) and the concentration of market power in the hands of a few platforms. Lastly, interviewees agreed that there is an urgent need for dialogue across the scholarly communication community to agree standards of behaviour in this area.

The 2017 ALLEA code says “Authors [should] ensure that their work is made available to colleagues in a timely, open, transparent, and accurate manner … and are honest in their communication to the general public and in traditional and social media.” The problem, however, is that this is an instruction to the author and not to the publisher or any third party host/disseminator of the work. In the section on “Research Misconduct or other Unacceptable Practices,” the code identifies as bad practice “Establishing or supporting journals that undermine the quality control of research.” However, it defines the scope of this bad practice as simply “predatory journals.”

The ALLEA code certainly attempts to bring within scope many areas of Open Science, but treats these subjects as issues pertaining to the author(s). This is an omission and, as this article has identified, a dangerous one if many users implicitly trust scholarly communication platforms. Standards which are expected of researcher(s) therefore do not explicitly cover publishers, hosters and disseminators of that research in the principal European code for research integrity.

Scholarly communication is an essential element of research: it supports rigorous professional conversation between researchers, with independent, critical thought at its core. Tracking the researchers’ interactions and persuading them to take certain actions will significantly diminish their genuine contribution to society. Research needs intuition, anticipation, hard work and designed serendipity. Being able to influence these elements, in both a transparent or covert manner, has the potential to control even further the course of human progress (in addition to the funding mechanisms). We need to avoid the unquestioning legitimisation of libertarian paternalism in scholarly communication (Thaler and Cass, 2003).

First of all, tracking and persuasive technologies could change the readership of a journal in a manner completely different than traditional editorial practices. Academic texts without proper editorial work could thrive based on the application of such technologies, instead of the quality of their conversation. Second, surveillance technologies used to build psychographic profiles, persuade algorithmically and pass as humans, pose the potential risk of influencing authors’ contributions, including research conclusions and recommendations. Even hypothesis generation could be influenced by the aforementioned technologies: for years there has been a quest to automate the identification of “hot” topics. This approach didn’t prove beneficial to research diversity or contribute to the development of generations of curious minds. Using AI and RPA for hypothesis refinement may represent an effective and efficient solution for researchers (The Royal Society and Alan Turing Institute, 2019), but not before defining what represents an ethical use of these technologies. Such systems “provide predictions, but no real insight. The “deep” learners are shallow indeed” (Carey, 2020).

Those we interviewed would welcome more evidence about tracking and persuasive technologies in scholarly communication. To produce such evidence, proper, well-resourced research is needed. This research needs to identify the actual use of those technologies, anticipate their potential use, but also determine which are the best approaches to engage with scholarly communication stakeholders in order to build a safe roadmap for the future. Early engagement is essential for steering a community in a smooth manner towards ethical developments.

The low number of expert opinions and the answers we received is another reasonable indication that we are acting at a frontier of human knowledge. These technologies are largely unknown and it is hard to determine how much priority they deserve.

We believe that in-depth research in this area would support practical approaches for Open Science. Such new understanding is key for at least two pillars of the new research culture: *The Future of Scholarly Communication* and *Research Integrity* (Lawrence and Mendez, 2020).

We believe that this is the best time to research the use of algorithmic technologies and their particular impact in scholarly communication. Furthermore, an advocacy and engagement programme is needed to connect stakeholders and agree on paths forward. The solution will be less about mandates; it will be about creating trust, encouraging transparency and building consensus.

## Recommendations for the Scholarly Communication Community

Both open science and scholarly communication communities need to widen their remit to include guidance and best practice on the use of tracking and persuading technologies. Research integrity codes such as the ALLEA code need significant revision to embrace these new areas. As the LERU Open Science Roadmap makes clear: “To embrace Open Science, universities and researchers need to embrace cultural change in the way they work, plan and operate. The result will infuse a culture of Open Science throughout the academic organisation and may support other evolutions in academic practice.” (Ayris et al., 2018b). The scope of such change needs to be as wide as possible, covering all players in the scholarly communications landscape.

Researchers need to be aware of the dangers associated with cookies. In this article, some of those questioned appreciated the benefits of tracking technologies. However, the findings of the quantitative and qualitative studies paint a concerning picture. There is little transparency and a significant potential for persuasive technologies to become commonplace. There is a need for education to enable researchers to understand the results of using dissemination and syndication platforms (including social media). Research funders, universities, publishers and tech companies should consider co-creating ethical requirements for such platforms. There also needs to be a global advocacy and awareness campaign to open up the issues around the use of cookies and trackers, highlighting the dangers as well as the benefits. This will help re-shape research culture at both national and international levels.

Open Science has also led to the unprecedented sharing of research data. While generally a positive change, this opens opportunities for the detrimental use of technology. An example is using data from a research project on human fears to train an algorithm that persuades people to buy insurance policies. For researchers and research organisations, including those that curate and maintain research datasets, it is important to be very conscious about what license should be granted to research data sets. Open Data is circulated in parallel and sometimes, instead of FAIR Data. These two concepts must not the confused with each other. While broader access and easier scrutiny to research data are necessary, the existence of malicious intent should be recognised and further development of creative commons models should be undertaken.

Our research data collection protocol was designed to use citizen scientists (volunteers) alongside researchers’ efforts. We also created short training materials to improve data collection, as the international community recommends. To attain the scale, diversity and geographical penetration of a full study, we think citizen science is a suitable approach for future work in this area as similar models exist (CSI-COP, 2021).

## Data Availability

The original contributions presented in the study are included in the article/Supplementary Materials, further inquiries can be directed to the corresponding author.

## Supplementary Material

The Supplementary Material for this article (including Further Recommended Readings) can be found online at: https://www.frontiersin.org/articles/10.3389/frma.2021.748095/full#supplementary-material

Data Sheet 1 Further Recommended Readings.
