# Supplementary material for: Perspectives on Open Science and The Future of Scholarly Communication: Internet Trackers and Algorithmic Persuasion
Source: Front Res Metr Anal. 2021 Dec 23;6:748095. doi: 10.3389/frma.2021.748095 (PMC8734967; doi:10.3389/frma.2021.748095)
Supplement: Supplementary file 2 [file DataSheet1.docx]

# Further Recommended Readings

Academia.edu. (2020). Privacy Policy. <https://www.academia.edu/privacy> [Accessed July 12, 2021].

AlgorithmWatch. (2021). AI Ethics Guidelines Global Inventory. AI Ethics Guidelines Global Inventory. <https://inventory.algorithmwatch.org/?sfid=172> [Accessed July 9, 2021].

Anderson, K. (2021a). Book Review: “The Lonely Century”. Thegeyser.Substack.Com. <https://thegeyser.substack.com/p/book-review-the-lonely-century> [Accessed July 9, 2021].

Anderson, K. (2021c). Part 2 - Edging Away from Utopianism. <https://thegeyser.substack.com/p/part-2-edging-away-from-utopianism> [Accessed July 12, 2021].

Anderson, K. (2021d). Part 3 - Swiss Cheddar. <https://thegeyser.substack.com/p/part-3-swiss-cheddar> [Accessed July 12, 2021].

Anderson, K. (2021e). Part 4 - Strange Bedfellows. <https://thegeyser.substack.com/p/part-4-duped-into-techno-utopianism> [Accessed July 12, 2021].

Anderson, K. (2018). Interpreting Elsevier's Acquisition of Aries Systems. <https://scholarlykitchen.sspnet.org/2018/08/06/interpreting-elseviers-acquisition-aries-systems/> [Accessed July 12, 2021].

Anderson, K. (2021f). Turning From The Internet’S False Idols. Thegeyser.Substack.Com. <https://thegeyser.substack.com/p/f988f734-b39a-4bd2-af30-ddc5838f47d3> [Accessed July 9, 2021].

Automated Team. (2020). Best Consent Management Platforms (CMPs) for Publishers. <https://headerbidding.co/best-consent-management-platforms/> [Accessed July 12, 2021].

Automated Team. (2020). A Comprehensive Post Third-Party Cookie Guide for Publishers. <https://headerbidding.co/survive-cookieless-era/> [Accessed July 12, 2021].

Automated Team. (2020). Customer Data Platform - Everything You Need to Know. <https://headerbidding.co/customer-data-platform/> [Accessed July 12, 2021].

Beerbaum, D. (2021). Artificial Intelligence Ethics Taxonomy - Robotic Process Automation (RPA) as Business Case. Special Issue ‘Artificial Intelligence& Ethics’ European Scientific Journal. doi:10.2139/ssrn.3834361.

Biometricks_Video. (2021). Vimeo. <https://vimeo.com/215391369> [Accessed July 9, 2021].

Boiten, E. (2021). Google's Scrapping Third-Party Cookies – but Invasive Targeted Advertising Will Live On. <https://theconversation.com/googles-scrapping-third-party-cookies-but-invasive-targeted-advertising-will-live-on-156530> [Accessed July 12, 2021].

Burgelman, J.C. (2021). Scholarly Publishing Needs Regulation - Research Professional News. <https://researchprofessionalnews.com/rr-news-europe-views-of-europe-2021-1-scholarly-publishing-needs-regulation/> [Accessed July 8, 2021].

Charikar, M. S. (2002). Similarity Estimation Techniques from Rounding Algorithms. Proceedings of the thirty-fourth annual ACM symposium on Theory of computing - STOC*.* doi:10.1145/509907.509965.

Crotty, D. (2021). New Open Access Business Models — What's Needed To Make Them Work?. <https://scholarlykitchen.sspnet.org/2021/04/28/new-open-access-business-models-whats-needed-to-make-them-work/> [Accessed July 12, 2021].

DORA. (2021). Home DORA. <https://sfdora.org/> [Accessed July 19, 2021].

Elsevier. (2018). Elsevier to acquire Aries Systems, a best-in-class publication workflow solutions provider. <https://www.elsevier.com/about/press-releases/corporate/elsevier-to-acquire-aries-systems-a-best-in-class-publication-workflow-solutions-provider> [Accessed July 12, 2021].

Elsevier. (2019). Privacy Policy. <https://www.editorialmanager.com/policies/elsevier/privacy-policy.html#InformationWeCollect> [Accessed July 12, 2021].

European Commission. (2021). What Does ‘Grounds Of Legitimate Interest’ Mean?. <https://ec.europa.eu/info/law/law-topic/data-protection/reform/rules-business-and-organisations/legal-grounds-processing-data/grounds-processing/what-does-grounds-legitimate-interest-mean_en#references> [Accessed July 09, 2021].

Extance, A. (2018). How AI Technology Can Tame The Scientific Literature. Nature 561 (7722): 273-274. doi:10.1038/d41586-018-06617-5.

Ferguson, C., Marcus, A., and Oransky, I. (2014). Publishing: The Peer-Review Scam. Nature 515 (7528): 480-482. doi:10.1038/515480a.

Fund, S. (2021). #Researchgate #Publishing #Academicpublishing. Linkedin.Com. <https://www.linkedin.com/posts/svenfund_researchgate-publishing-academicpublishing-activity-6764902980761243649-Ecqo> [Accessed July 9, 2021].

Gabriel, A. (2020). Artificial Intelligence in Scholarly Communications: An Elsevier Case Study. Information Services & Use. 39:4. doi:10.3233/isu-190063.

Gallo, C. (2019). IBM's AI Machine Makes A Convincing Case That It's Mastering The Human Art Of Persuasion. Forbes. <https://www.forbes.com/sites/carminegallo/2019/01/13/ibms-ai-machine-makes-a-convincing-case-that-its-mastering-the-human-art-of-persuasion/> [Accessed July 9, 2021].

Gershgorn, D. (2021). The FTC Forced A Misbehaving A.I. Company To Delete Its Algorithm. Medium. <https://onezero.medium.com/the-ftc-forced-a-misbehaving-a-i-company-to-delete-its-algorithm-124d9f7e0307> [Accessed July 9, 2021].

Guglielmi, G. (2020). The Next-Generation Bots Interfering With The US Election. Nature 587 (7832): 21-21. doi:10.1038/d41586-020-03034-5.

Hawkins, E., Hofmayer, S., Noyes, D., Schoenenberger, H. and Winter, S. (2020). Researchers At The Centre: Content Discoverability, Visibility, And Access: An Evaluation Of The Content Syndication Partnership Between Springer Nature And Researchgate. Ebook. Springer Nature. https://media.springernature.com/full/springer-cms/rest/v1/content/18300962/data/v4.

Heikkila, M. (2021). Europe Eyes Strict Rules For Artificial Intelligence. <https://www.politico.eu/article/europe-strict-rules-artificial-intelligence/> [Accessed July 12, 2021].

HighWire. (2018). The Potential Impact of Artificial Intelligence on the Scholarly Publishing Ecosystem. <https://www.highwirepress.com/insight/the-potential-impact-of-artificial-intelligence-on-the-scholarly-publishing-ecosystem/> [Accessed July 12, 2021].

Hinchliffe, L. J. (2021). Twitter post. <https://twitter.com/lisalibrarian/status/1385646781283831812> [Accessed July 12, 2021].

IBM Research. (2018). AI Learns the Art of Debate. Youtube. <https://youtu.be/UeF_N1r91RQ> [Accessed July 9, 2021].

ICO. (2021). How Do We Apply Legitimate Interests In Practice? <https://ico.org.uk/for-organisations/guide-to-data-protection/guide-to-the-general-data-protection-regulation-gdpr/legitimate-interests/how-do-we-apply-legitimate-interests-in-practice/> [Accessed July 08, 2021].

ICO. (2021). What Is The ‘Legitimate Interests’ Basis?. <https://ico.org.uk/for-organisations/guide-to-data-protection/guide-to-the-general-data-protection-regulation-gdpr/legitimate-interests/what-is-the-legitimate-interests-basis/> [Accessed July 9, 2021].

IESC, Admin M2. (2018). Artificial Intelligence, Machine Learning, and Deep Learning: Same Context, Different Concepts. <https://master-iesc-angers.com/artificial-intelligence-machine-learning-and-deep-learning-same-context-different-concepts/> [Accessed July 12, 2021].

Irwin, L. (2020). The GDPR: Legitimate Interest – What Is It And When Does It Apply? - IT Governance Blog En. <https://www.itgovernance.eu/blog/en/the-gdpr-legitimate-interest-what-is-it-and-when-does-it-apply> [Accessed July 8, 2021].

Isabel Prade. (2021). Biometricks. <https://isabelprade.com/biometricks> [Accessed July 9, 2021].

Isabel Prade. (2021). Projects. <https://isabelprade.com/projects> [Accessed July 9, 2021].

Jatain, V. (2021). What Digital Publishers Need To Know About Cookie Blocking. Adage.Com. <https://adage.com/article/industry-insights/what-digital-publishers-need-know-about-cookie-blocking/2228046> [Accessed July 9, 2021].

Joint Council for the Welfare of Immigrants. (2020). We Won! Home Office to Stop Using Racist Visa Algorithm. <https://www.jcwi.org.uk/news/we-won-home-office-to-stop-using-racist-visa-algorithm> [Accessed July 12, 2021].

Kelion, L. (2021). Spy Pixels In Emails Have Become Endemic. BBC News. <https://www.bbc.com/news/technology-56071437> [Accessed July 9, 2021].

Kosinski, M., D. Stillwell, and T. Graepel. (2013). Private Traits and Attributes Are Predictable from Digital Records of Human Behavior. Proceedings of the National Academy of Sciences. 110:15. doi:10.1073/pnas.1218772110.

Kraker, P. (2021). Open Search Tools Need Sustainable Funding - Research Professional News. <https://www.researchprofessionalnews.com/rr-news-europe-views-of-europe-2021-5-open-search-tools-need-sustainable-funding/> [Accessed July 8, 2021].

Krishna, A. (2018). AI Learns The Art Of Debate With IBM Project Debater. IBM Research Blog. <https://www.ibm.com/blogs/research/2018/06/ai-debate/> [Accessed July 9, 2021].

Lanier, J., and Weyl. E.G. (2018). A Blueprint For A Better Digital Society. Harvard Business Review. <https://hbr.org/2018/09/a-blueprint-for-a-better-digital-society> [Accessed July 9, 2021].

Larson, E.J. (2021). The Myth of Artificial Intelligence: Why Computers Cant Think the Way We Do. Cambridge, MA: Belknap Press of Harvard University Press.

Ledford, H. (2020). How Facebook, Twitter And Other Data Troves Are Revolutionizing Social Science. Nature 582 (7812): 328-330. doi:10.1038/d41586-020-01747-1.

LERU. (2016). Citizen Science At Universities: Trends, Guidelines And Recommendations. Ebook. LERU. https://www.leru.org/files/Citizen-Science-at-Universities-Trends-Guidelines-and-Recommendations-Full-paper.pdf.

Markoff, J. (2020). A Case For Cooperation Between Machines And Humans. Nytimes.Com. <https://www.nytimes.com/2020/05/21/technology/ben-shneiderman-automation-humans.html> [Accessed July 9, 2021].

Milliken, A. (2021) Home Office Algorithm to Detect Sham Marriages May Contain Built-in Discrimination. <https://www.thebureauinvestigates.com/stories/2021-04-19/home-office-algorithm-sham-marriages> [Accessed July 12, 2021].

Mirowski, P. (2018). The Future(S) Of Open Science. Social Studies Of Science 48 (2): 171-203. doi:10.1177/0306312718772086.

Naudé, W. (2021). Artificial Intelligence: Governments See Huge Business Potential, But Ignore The Downsides. The Conversation. <https://theconversation.com/artificial-intelligence-governments-see-huge-business-potential-but-ignore-the-downsides-164645>.

Naughton, J. (2021). The BBC’S Interviewer Found Himself On A Sticky Wicket With Google’S CEO. The Guardian. <https://www.theguardian.com/commentisfree/2021/jul/17/the-bbcs-interviewer-found-himself-on-a-sticky-wicket-with-googles-ceo> [Accessed July 22, 2021].

Nordling, L. (2021). Give African Research Participants More Say In Genomic Data, Say Scientists. Nature 590 (7847): 542-542. doi:10.1038/d41586-021-00400-9.

O'Brien, T. L. (2021). Google Puts Lid on Cookie Jar and Ends an Internet Era | Commentary. <https://www.seattletimes.com/business/technology/google-puts-lid-on-cookie-jar-and-ends-an-internet-era-commentary/> [Accessed July 12, 2021].

Papakyriakopoulos, O., Hegelich S., Shahrezaye M., Serrano, J.C.M. (2018). Social Media And Microtargeting: Political Data Processing And The Consequences For Germany. Big Data & Society 5 (2): 205395171881184. doi:10.1177/2053951718811844.

ResearchGate. (2021). Privacy Policy. <https://www.researchgate.net/privacy-policy> [Accessed July 12, 2021].

Retraction Watch. (2021). Elsevier Journals Ask Retraction Watch To Review COVID-19 Papers. <https://retractionwatch.com/2021/03/09/elsevier-journals-ask-retraction-watch-to-review-covid-19-papers/> [Accessed July 9, 2021].

Rinke, E., M. (2016). The Impact of Sound-Bite Journalism on Public Argument. Journal of Communication. 66:4. doi:10.1111/jcom.12246.

Schonfeld, R. (2021). Publishers Still Don't Prioritize Researchers. The Scholarly Kitchen. <https://scholarlykitchen.sspnet.org/2021/01/26/publishers-fail/> [Accessed July 9, 2021].

Sciencedaily. (2021). Robots Appear More Persuasive When Pretending To Be Human: When Bots Disclose Their Non-Human Nature, Their Efficiency Is Compromised". <https://www.sciencedaily.com/releases/2019/11/191112113952.htm> [Accessed July 9, 2021].

Smith, A. (2021). Using Artificial Intelligence And Algorithms. Federal Trade Commission. <https://www.ftc.gov/news-events/blogs/business-blog/2020/04/using-artificial-intelligence-algorithms> [Accessed July 9, 2021].

Sparkes, M. (2021). Can The European Union Prevent An Artificial Intelligence Dystopia?. <https://www.newscientist.com/article/2274720-can-the-european-union-prevent-an-artificial-intelligence-dystopia/> [Accessed July 12, 2021].

Springer Nature Group. (2021). Springer Nature And Researchgate To Move Forward With Long-Term Content-Sharing Partnership. <https://group.springernature.com/de/group/media/press-releases/springer-nature-researchgate-move-forward-with-long-term-partner/18357842> [Accessed July 22, 2021].

Stein, G., Rich, T., Verdin, Z., & Ahearn, C. (2021). Clarivate, ProQuest, and our Resistance to Commercializing Knowledge. Commonplace. <https://doi.org/10.21428/6ffd8432.fb0245ff>

STM - International Association of STM Publishers. (2020). Eefke STM 2020 presentatie Finished Version 3 2m19s 1. Youtube. <https://youtu.be/TBbZuZ2Vm7g> [Accessed July 9, 2021].

STM. (2021). Tech Trends 2024 - STM <https://www.stm-assoc.org/standards-technology/stm-tech-trends-2024-focus-on-the-user-connect-the-dots/> [Accessed July 9, 2021].

Stop Tracking Science. (2021). Stop Tracking Science. <https://stoptrackingscience.eu/> [Accessed July 12, 2021].

The Economist. (2021). The New Rules Of The “Creator Economy”. (2021). <https://www.economist.com/briefing/2021/05/08/the-new-rules-of-the-creator-economy> [Accessed July 9, 2021].

The University Of Edinburgh. (2021).CAMARADES. <https://www.ed.ac.uk/clinical-brain-sciences/research/camarades> [Accessed July 19, 2021].

The Royal Society, and The Alan Turing Institute. (2019). The AI Revolution in Scientific Research. <https://royalsociety.org/-/media/policy/projects/ai-and-society/AI-revolution-in-science.pdf>.

UCL Department Of Geography. (2021). UCL's Interdisciplinary Extreme Citizen Science Research Group. <https://www.geog.ucl.ac.uk/research/research-centres/excites/about-us> [Accessed July 22, 2021].

Upshall, M. (2019). Using AI to Solve Business Problems in Scholarly Publishing. Insights 32 (1): 13. DOI: <http://doi.org/10.1629/uksg.460>

WhitePress. (2021). WhitePress - Content Marketing platform. <https://www.whitepress.net/> [Accessed July 12, 2021].

Zhou, H., and Prosser, M. (2021). Guest Post — Bioasq For The Win: Inside The Healthiest Competition You’Ve Never Heard Of. The Scholarly Kitchen. <https://scholarlykitchen.sspnet.org/2021/06/10/guest-post-bioasq-for-the-win-inside-the-healthiest-competition-youve-never-heard-of/> [Accessed July 9, 2021].
